# Exosomes Regulate the Epithelial–Mesenchymal Transition in Cancer

**DOI:** 10.3389/fonc.2022.864980

**Published:** 2022-03-14

**Authors:** Jingwen Jiang, Jiayu Li, Xiumei Zhou, Xueqin Zhao, Biao Huang, Yuan Qin

**Affiliations:** College of Life Sciences and Medicine, Zhejiang Sci-Tech University, Hangzhou, China

**Keywords:** cancer, epithelial–mesenchymal transition, exosome, tumor metastasis, contents in exosomes

## Abstract

Exosomes are important mediators of intercellular communication and participate in complex biological processes by transferring a variety of bioactive molecules between cells. Epithelial–mesenchymal transition (EMT) is a process in which the cell phenotype changes from epithelioid to mesenchymal-like. EMT is also an important process for cancer cells by which they acquire invasive and metastatic capabilities, which aggravates the degree of tumor malignancy. Numerous studies have demonstrated that exosomes encapsulate various components, such as microRNAs and proteins, and transfer information between tumor cells or between tumor cells and the tumor microenvironment, thereby regulating the EMT process. Exosomes can also be used for cancer diagnosis and treatment or as a drug delivery platform. Thus, they can be used as a therapeutic tool to control the occurrence of EMT and affect cancer progression. In this review, we summarize the latest research advancements in the regulation of the EMT process in tumor cells by the contents of exosomes. Furthermore, we discuss the potential and challenges of using exosomes as a tool for cancer treatment.

## Introduction

Malignant tumors are complicated structures composed of cancer cells and tumor stromal cells, such as fibroblasts, immune cells, and epithelial cells. These tumor stromal cells continuously release a variety of cytokines and active substances, which directly or indirectly affect the tumor microenvironment (TME), thereby affecting the tumor cells themselves and the nearby normal cells. Tumor-derived exosomes (TDEs) are one of the tools for the interchange of substances between tumor cells and the TME. The main physiological role of TDEs is to mediate cell–cell communication by transferring small RNAs, such as microRNAs (miRNAs) (1), long noncoding RNAs (lncRNAs) (2), proteins (3), DNAs, and messenger RNAs (mRNAs) (4).

Metastasis significantly increases cancer malignancy. Before metastasis, a pre-metastasis niche (PMN) is established in the target organ, providing a suitable microenvironment to support the colonization of metastatic tumor cells (5). A large number of studies have shown that TDEs are involved in the formation of PMNs by inducing vascular permeability and angiogenesis, activating fibroblasts, and promoting inflammation (6–9). TDEs also mediate intercellular communication and play an essential role in epithelial–mesenchymal transition (EMT). EMT is a reversible process in which cell morphology transforms from the epithelial state into the mesenchymal state; it is a key process before tumor cells migrate and invade. During EMT, E-cadherin expression in tumor cells is decreased, resulting in decreased adhesion and loss of apical polarity and basal anchoring, which enable tumor cells to easily leave the primary lesion and migrate to other organs (10). TDEs carry metabolites and signaling molecules from donor organs and are taken up by target organ cells through endocytosis. These contents activate intracellular EMT-related signaling pathways and promote the occurrence of EMT (1, 11–13).

In this review, we summarize the effects of different exosome contents on tumor cell metastasis, particularly EMT, and describe the related molecular mechanisms. Furthermore, we present the current research progress in the treatment of cancer with exosomes and discuss the prospects and potential challenges of using exosomes as therapeutic tools for cancer treatment.

## Exosomes

Exosomes are extracellular vesicles (EVs) with a lipid bilayer (30–100 nm in diameter) that arise from the luminal membranes of multivesicular bodies (MVBs) and are released into the extracellular matrix after MVBs fuse with the cell membrane (14). Exosomes are produced by many cell types, including T cells (15), B cells (16), epithelial cells (17), and tumor cells (18). In 1987, Johnstone et al. (19) first isolated these extracellular vesicles from an *in vitro* culture of sheep reticulocytes and named them “exosomes”. However, exosomes were largely ignored because they were initially considered cellular “garbage bags”. In 1996, exosomes were found to play a role in antigen presentation during T-cell responses restricted by B lymphocyte-induced antigen-specific MHC class II (20). Since then, exosomes have begun to be appreciated for their active function in intercellular communication.

Exosomes contain a variety of bioactive molecules, some of which are related to their physiological functions. For example, some proteins are involved in exosome biogenesis (TSG101, flotillin, and Alix), MVB transformation, and exosome release. Tetraspanins (CD9, CD63, CD81, and CD82) and heat shock proteins (Hsp90 and Hsp70) are involved in exosome transport and membrane fusion with target cells (21–23). Some of these proteins have been used as markers for exosome detection (TSG101, Hsp70, and CD63).

TDE-mediated long-distance intercellular communication plays a key role in cancer occurrence and development. Increasing evidence indicates that TDEs regulate the TME and promote cancer cell proliferation, angiogenesis, EMT generation, and PMN formation (3, 7, 24, 25). However, not all news about TDEs is negative. Recently, the modification of exosomes as a cancer treatment tool has become a hot research topic. In addition to investigating the use of exosomes for cancer diagnosis and prognostic analysis (26), researchers examined the use of exosomes for EMT reversal and drug delivery (27, 28). Notably, animal cells are not the only cells that produce exosomes. Plant cells also produce similar extracellular vesicles, called plant exosome-like nanovesicles (PELNVs), which have received increasing attention as natural drug delivery nanoplatforms (29).

## EMT in Cancer Cells

In the 1970s, Hay first observed the EMT process during embryonic development and proposed the concept of EMT (30). Subsequently, the roles of EMT in embryonic development, gastrulation, organ development, and maturation were discovered one after another (31). EMT is a process in which cell morphology changes from the epithelial state to the mesenchymal state. This process also occurs in cancer cells.

EMT is usually accompanied by tumor occurrence, invasion, metastasis, and resistance to therapy. Recent studies have shown that EMT occurs in different cellular states and is not a binary process (32–35). In monolayer cultures, epithelial cells are polygonal in shape with apical polarity and are closely connected to each other into sheets. In contrast, mesenchymal cells are spindle-shaped, lose the polarity of the apical group, and loosely adhere to the extracellular matrix. Hence, mesenchymal cells have better mobility and invasion ability than epithelial cells, and tumor cells are more prone to vascular infiltration and metastasis after EMT.

**Figure 1:**
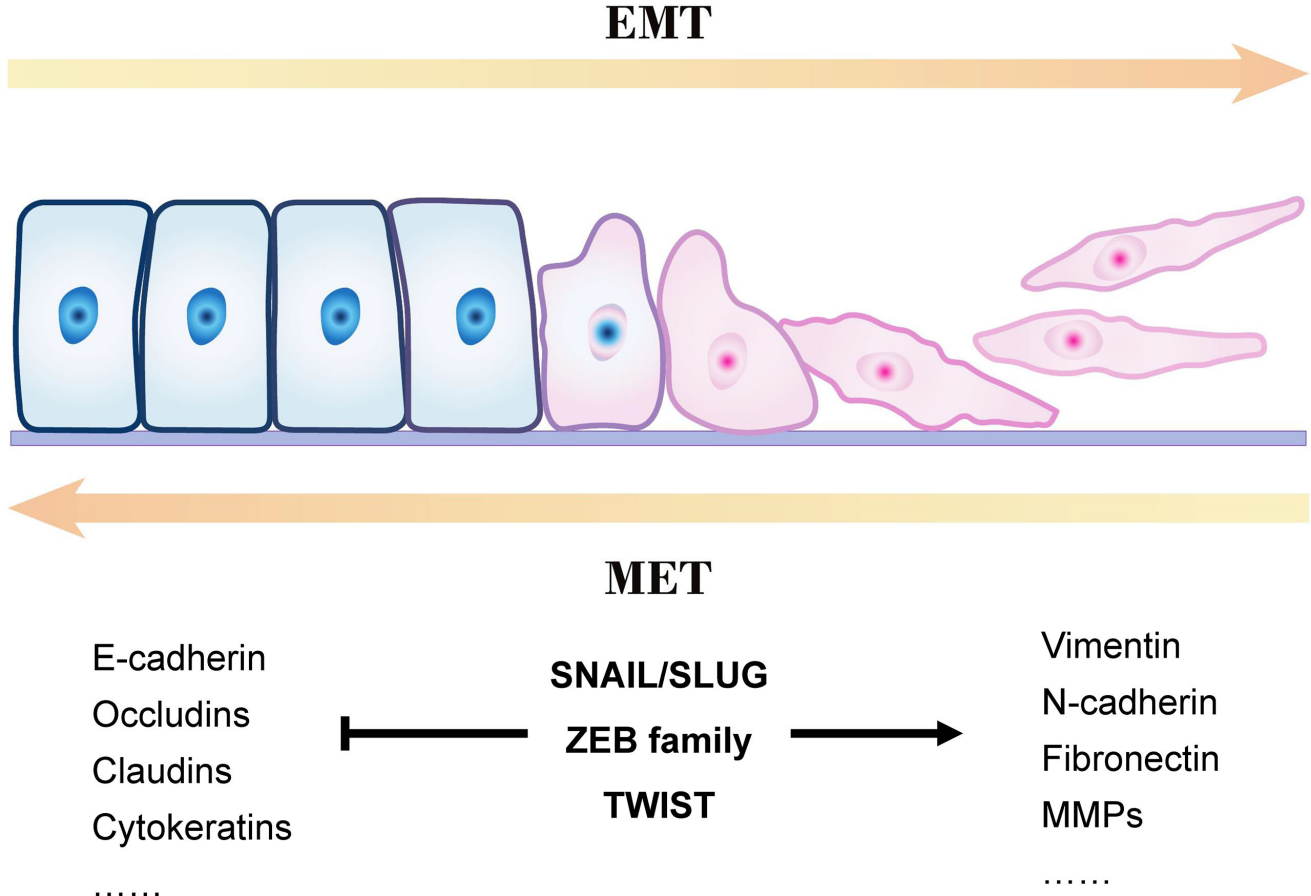
Outline of a typical epithelial–mesenchymal transition (EMT) program.

**Figure 1** presents an overview of the EMT process. Epithelial cells are connected by tight junctions and adhesion junctions. Molecules involved in establishing the epithelial cell state also play an essential role in maintaining cell polarity. When the expression of genes involved in maintaining the cell state is changed in epithelial cells, their cellular properties are gradually lost and they gradually acquire mesenchymal properties.

EMT is a reversible process, in contrast to mesenchymal–epithelial transition (MET), but it is not a bipolar process; that is, the cells are either in the epithelial or mesenchymal state. E-cadherin is a membrane protein that increases cell–cell adhesion, and its high expression is a marker of epithelial cells. However, mesenchymal cells often lack E-cadherin and express vimentin and N-cadherin (36, 37). Studies have demonstrated co-expression of E-cadherin, N-cadherin, and vimentin in some cells during EMT, indicating that these cells are between the epithelial and mesenchymal states (called hybrid E/M cells) (38–40). This phenomenon proves that EMT (or MET) is not a binary process; it occurs gradually, and cells with different phenotypes can transform into one another.

EMT is widely considered as a key process in the generation of cancer stem cells (CSCs). EMT occurs after non-CSCs are stimulated by EMT-inducing signals, which induce the expression of cell stemness markers CD133 and CD44. As a result, these cells gain self-renewal capability, the invasion–metastasis cascade is activated, tumor metastasis is promoted *in vivo*, and even drug resistance develops (41–44). Exosomes often act as “accomplices” in this process by facilitating the communication between cells and the extracellular matrix to complete the EMT process and convert more non-CSCs to CSCs, accelerating the progression of cancer deterioration (13, 45).

## Exosomes Carry EMT-Related Active Molecules

**Figure 2:**
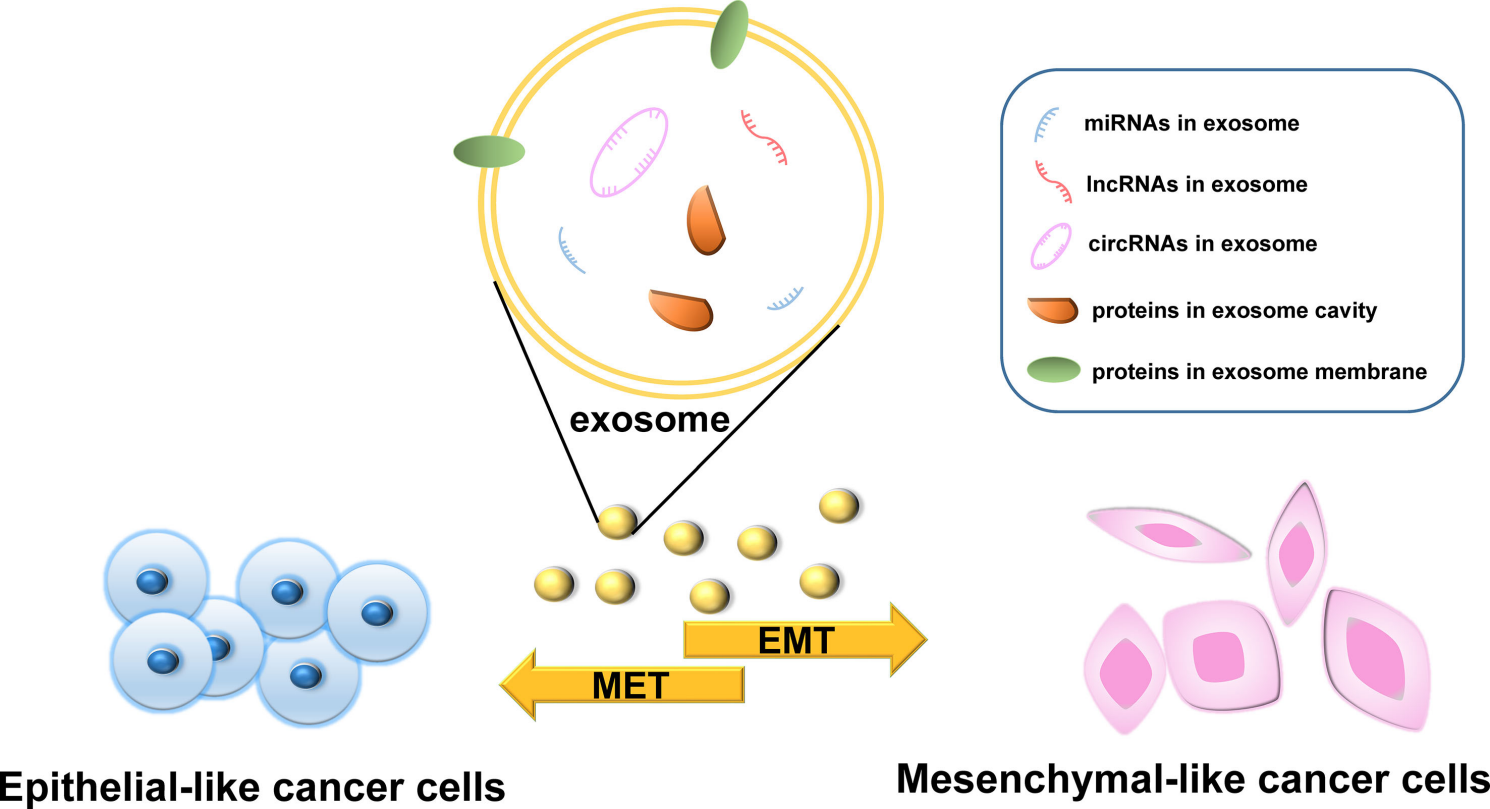
The effects of molecules carried by exosomes on epithelial–mesenchymal transition (EMT).

The various bioactive molecules carried by exosomes have different regulatory effects on the EMT process in cancer and different underlying mechanisms. In this section, we describe the four main types of molecules carried by exosomes, namely, proteins, microRNAs (miRNAs), long non-coding RNAs (lncRNAs), and circular RNAs (circRNAs), and discuss those that have been studied the most (**Figure 2**). However, the active components in exosomes are not limited to the biomolecules listed here; other components need to be further studied.

### Exosomal Proteins and Peptides

Exosomes contain various proteins and peptides, some of which are enclosed in the exosome cavity or membrane (**Table 1**).

**Table 1:** The effects of proteins carried by exosomes on EMT.

Proteins	Cancer Type	Effect	Reference
Snail1	Lung cancer	Inhibit E-cadherin expression, induce the occurrence of EMT.	(46)
HSP gp96, HIF-1	Breast cancer	Degrade p53, increases therapy resistance and accelerate EMT.	(47)
MMP-13	NPC	Upregulate vimentin, down-regulate E-cadherin, promote EMT.	(48)
IL-6	Bladder cancer	Increase pSTAT3 and pAKT level, activate the STAT3 signaling pathway, promote EMT.	(3)
MAP-17	Breast cancer	Interact with NUMB protein, activate the Notch signaling pathway, increase secretion of extracellular vesicles, promote the horizontal transmission and metastasis of EMT.	(49)
ITGBL1	CRC	Combine to TNFAIP3 and activate the NF-κB signaling pathway, convert fibroblasts into CAFs, promote the formation of PMN and the generation of EMT.	(50)
PSGR	PC	mediate the enrichment of mRNA in exosomes in EMT-related pathways to facilitate EMT.	(51)
Intergrin(α_6_β_4,_ α_6_β_1,_ α_V_β_5._etc)		Determine the target organs of exosomes.	(52)

Transcription factors (TFs) can be bind specific gene sequences to control the expression of target genes. Members of the zinc finger transcription factor Snail superfamily often “travel” between cells in exosomes. Cancer-associated fibroblast (CAF)-derived exosomes carry Snail1 to inhibit E-cadherin expression and thus induce the occurrence of EMT in A549 lung cancer cells (46). SLUG and SOX2 are significantly upregulated in exosomes secreted from thyroid CSCs and can initiate EMT programming in recipient cells (2). In addition to members of the Snail superfamily, other common TFs are found in exosomes, serving as a “bridge” for tumor cells to interact with the TME. For example, under hypoxic conditions, paclitaxel-resistant breast cancer (PR-BC) cells upregulate the expression of HSP gp96. Then, the overexpressed HSP gp96 and hypoxia-inducible factor (HIF-1) are transported to paclitaxel-sensitive breast cancer (PS-BC) cells through exosomes to degrade p53, increase PS-BC resistance, and accelerate EMT (47). In nasopharyngeal carcinoma (NPC) cells, hypoxic exosomes carry MMP-13, which significantly upregulates the expression of vimentin in recipient cells and reduces the level of E-cadherin, thereby promoting the EMT of NPC cells and enhancing their migratory and invasive abilities (48).

The exosomal cavity also contains proteins and TFs. A previous study confirmed that CAF-derived exosomes can promote EMT in non-invasive bladder cancer cells and transform these cells into an aggressive phenotype by secreting IL-6 (3). A small protein, MAP17, is transferred between subsets of tumor cells by TDEs to promote the horizontal transmission of metastasis and EMT (49). Ji et al. (50) found that exosomal integrin beta-like 1 (ITGBL1) from primary tumors can convert fibroblasts in distal organs into CAFs by combining with TNFAIP3 and activating the NF-κB signaling pathway, thereby promoting the formation of PMNs and EMT. As previously mentioned, TFs participate in the information exchange between tumor cells and the TME through exosomes. This, however, is not a privilege of TFs. Breast cancer cells overexpress survivin (member of the inhibitor of apoptosis protein family) and secrete it into the extracellular environment through exosomes. CAFs then internalize the exosomes, upregulate the expression of SOD1, and transform into myofibroblasts, which in turn promote breast cancer cell proliferation, EMT, and stem cell formation (53).

Some proteins are present in the exosomal membrane. Prostate-specific G-protein coupled receptor (PSGR) is overexpressed in prostate cancer (PC) cells and can spread to surrounding cells along with TDEs, thereby mediating the enrichment of mRNA in exosomes in EMT-related pathways and facilitating the migration, invasion, stem cell differentiation, and EMT of PC cells and normal prostate epithelial cells (51). Proteins located in the exosomal membrane play a role in determining the target organs for metastasis during tumor migration and EMT. For example, exosomal integrins α_6_β_4_ and α_6_β_1_ target lung metastasis, whereas exosomal integrin α_V_β_5_ is associated with liver metastasis (52).

Moreover, peptides with a smaller molecular weight than that of proteins can also be delivered to target cancer cells. Exosomes modified to secrete tumor necrosis factor-related apoptosis-inducing ligand (TRAIL) have remarkable efficacy in inducing cancer cell apoptosis (54). Survivin has anti-apoptotic effects in many types of cancers. Exosomes that transfer mutated survivin (T34A) can promote apoptosis in pancreatic cancer (55). In the presence of antigen-presenting cells, dendritic cell-derived exosomes load different types of peptide antigens (e.g., major histocompatibility complex class I and class II) and then stimulate T cells to participate in the anti-tumor response (56). However, the regulation of EMT in tumor cells by peptides carried by exosomes has rarely been reported. However, there is a reasonable prospect that more advances in this research field can be made in the near future.

### Exosomal MiRNAs

MiRNAs, noncoding RNAs that contain 20–24 nucleotides, are the most widely studied molecules in exosomes (**Table 2**). They cannot be translated into proteins, but they regulate gene expression at the post-transcriptional and translational levels by binding to mRNA. Under the protection of exosomes, exosomal miRNAs can avoid digestion by ribonucleases and are more stable. Thus, they can safely reach the target organ through the systemic circulation (61). However, different exosomal miRNAs have opposite effects, namely, promotion or inhibition of the tumor EMT process.

**Table 2:** The effects of miRNAs carried by exosomes on EMT.

miRNAs	Cancer Type	Effect	Reference
miR-92a-3p	HCC	inhibit the expression of the tumor-suppressor gene PTEN, activate the Akt/Snail signaling pathway, facilitates the occurrence of EMT.	(57)
	CRC	activate the Wnt/β-catenin signaling pathway, inhibit mitochondrial apoptosis by inhibiting FBXW7 and MOAP1, helping CRC cells to obtain stemness and promote EMT.	(12)
miR-181d-5p	Breast cancer	Reduce CDX2 expression, inhibit the expression of HOXA5, thereby promoting EMT.	(11)
miR-375-3p	Colon cancer	promote E-cadherin expression, downregulate the expression of vimentin, inhibit EMT, and promote MET	(58)
miR-34a-5p	CSCC	Binding to AXL, reduce the activity of β-catenin, inhibit EMT.	(59)
miR-253p, miR-130b-3p, miR-425-5p, miR-934	CRC	Target macrophages, downregulate PTEN expression, activate the PI3K/Akt signaling pathway to induce the M2 polarization of TAMs. M2-polarized TAMs enhance EMT, secrete BLC to induce PMN formation and promote CRC liver metastasis.	(8, 60)

Exosomal miR-92a-3p is highly expressed in hepatocellular carcinoma (HCC) and colorectal cancer (CRC) (12, 57). In HCC, exosomal miR-92a-3p inhibits the expression of the tumor suppressor gene PTEN, activates the Akt/Snail signaling pathway, and facilitates EMT and metastasis (57). In CRC, the increased expression of miR-92a-3p activates the Wnt/β-catenin signaling pathway and inhibits mitochondrial apoptosis by inhibiting FBXW7 and MOAP1, thereby helping CRC cells obtain stemness and promote EMT and metastasis (12). Exosomal miR-181d-5p targets the transcription factor CDX2 in breast cancer, thereby inhibiting the expression of HOXA5 and promoting cancer cell proliferation, invasion, metastasis, and EMT (11).

The expression of some miRNAs in exosomes negatively correlates with the degree of EMT. Tumor-derived exosomal miR-375-3p promotes E-cadherin expression, downregulates vimentin expression, inhibits EMT, and promotes MET (58, 62). In addition, miR-34a-5p expression is reduced in CAF-derived exosomes and can be transferred to oral squamous cell cancer (CSCC) cells by exosomes from fibroblasts. However, following miR-34a-5p overexpression in CAFs, exosomal miR-34a-5p can reduce the activity of β-catenin by binding to its downstream target AXL, thereby inhibiting EMT in CSCC cells (59).

Exosomal miRNAs can also “incite” macrophages as accomplices in EMT. There are two subtypes of tumor-associated macrophages (TAMs): M1 and M2. The pro-tumorigenic M2 subtype can restrain T-cell function and promote tumor immune escape (63, 64). Exosomes from CRC cells transport miR-253p, miR-130b-3p, miR-425-5p, and miR-934 to macrophages. These miRNAs downregulate PTEN expression and activate the PI3K/Akt signaling pathway to induce the M2 polarization of TAMs. M2-polarized TAMs enhance EMT (60) and secrete B lymphocyte chemoattractant (BLC) to induce PMN formation and promote CRC liver metastasis (8).

### Exosomal LncRNAs

LncRNAs, belonging to the noncoding RNA family, are also commonly present in exosomes (**Table 3**). In contrast to miRNAs, lncRNAs are more than 200 kb in length, less conserved across species, and have higher tissue-specificity (70).

**Table 3:** The effects of lncRNAs carried by exosomes on EMT.

LncRNAs	Cancer Type	Effect	Reference
LINC00659	CRC	Bind to tumor suppressor miR-342-3p, enhance the ANXA2 expression, promote cancer development.	(65)
LncRNA RPPH1	CRC	Influence the occurrence of EMT induced by the tumor microenvironment	(66)
HOTTIP	GC	Activate HMGA1, cause the GC cells to undergo the EMT process and acquire cisplatin resistance.	(67)
LncRNA-Sox2ot	PADC	Regulates Sox2 expression and promotes PADC metastasis, invasion and EMT	(68)
MALAT1, linc-ROR	Thyroid cancer	Secreted by CSC, induce non-cancer thyroid cells to produce EMT	(2)
LINC00960, LINC02470	Bladder cancer	upregulate the β-catenin signaling pathway, the Notch signaling pathway, and the Smad2/3 signaling pathway, activate EMT process and promote deterioration.	(69)

In CRC, CAF-derived LINC00659 directly binds to the tumor suppressor miR-342-3p in cancer cells, enhances the expression of ANXA2, which is involved in the EMT process, and promotes the development of CRC (65). LncRNA RPPH1 can also induce EMT in CRC cells. It binds to TUBB3 to prevent ubiquitination and induces EMT by affecting the TME (66). In gastric cancer (GC) cells, the exosomal lncRNA HOTTIP activates its target HMGA1, causing GC cells to undergo EMT and acquire cisplatin resistance (67). In pancreatic ductal adenocarcinoma (PADC), exosomal lncRNA-Sox2ot competitively binds to the miR-200 family, affecting Sox2 expression and resulting in changes in PADC metastasis, invasion, and EMT (68).

LncRNAs also act in a hierarchical order. Hardin et al. (2) found that exosomes secreted by thyroid CSCs can carry lncRNA MALAT1 and linc-ROR as “infection factors” and induce EMT in non-cancerous thyroid cells. Highly malignant bladder cancer cells transmit LINC00960 and LINC02470 to early bladder cancer cells through exosomes, thereby inducing further deterioration and activating the EMT process by upregulating the β-catenin, Notch, and Smad2/3 signaling pathways (69).

### Exosomal CirRNAs

CircRNAs act as competing endogenous RNAs (ceRNAs) in many tumors by capturing miRNAs (71). Similar to other noncoding RNAs, exosomal circRNAs can also play different roles in different tumor cells (**Table 4**).

**Table 4:** The effects of circRNAs carried by exosomes on EMT.

CircRNAs	Cancer Type	Effect	Reference
circ_0001359	PC	Capture miR-582-3p to activate the TGF-β signaling pathway, promote EMT.	(72)
circPRMT5	UCB	capture miR-30c, regulate the Snail1/E-cadherin signaling pathway, promote EMT.	(73)
circ007293	PTC	inhibit the activity of miR-653-5p to upregulate the expression of paired box 6, accelerate EMT	(74)
circNRIP1	GC	Promotes GC metastasis and upregulates EMT markers in GC cells	(71)
circFARSA	NSCLC	induce polarization of macrophages toward the M2 phenotype, thereby promoting EMT.	(75)
circPTPRA	NSCLC	stimulate mir-96-5P to inhibit metastasis and EMT	(76)
circ-CPA4	NSCLC	Targeting the let-7 miRNA/PD-L1 axis to modulate the mobility and EMT of NSCLC cells	(77)

In PC, exosomal circ_0001359 activates the TGF-β signaling pathway by capturing miR-582-3p and promoting EMT in RWPE-1 cells (72). CircRNAs also play a significant role in EMT in bladder cancer. In urothelial carcinoma of the bladder (UCB), the expression of exosomal circPRMT5 is upregulated to capture miR-30c, regulate the Snail1/E-cadherin signaling pathway, and promote EMT in UCB cells (73). In papillary thyroid carcinoma (PTC), exosomal circ007293 inhibits the activity of miR-653-5p by trapping miR-653-5p, thereby upregulating the expression of paired box 6 in PTC cells and accelerating the EMT of tumor cells (74). Exosomes can also deliver circNRIP1, promote GC metastasis *via* EMT, and upregulate EMT markers in GC cells (71). circRNAs affect the progression of non-small cell lung carcinoma (NSCLC) *via* multiple pathways. For example, exosomal circFARSA secreted by NSCLC cells induces the polarization of macrophages toward the M2 phenotype, thereby promoting EMT (75). Exosomal circPTPRA can stimulate mir-96-5P to inhibit metastasis and EMT in NSCLC cells (76). In addition, Circ-CPA4 can affect the migration and EMT of NSCLC cells by targeting the let-7 miRNA/PD-L1 axis (77). Thus, circRNAs can function not only as oncogenes but also as tumor suppressors.

## Exosomes as a Therapeutic Tool for EMT

Exosomes can transport contents between cells and protect them from degradation, making them more suitable for material delivery than liposomes (78). In a study by SS et al. (79), miR-381-3p mimics were encapsulated in ADMSC-exosomes using electroporation. Scratch assays and cell invasion experiments showed that the ADMSC-exosomes wrapped with miR-381-3p could target the Wnt signaling pathway and EMT transcription factors. This reduced the metastatic and invasive abilities of the TNBC cells. TDEs can also be used as vectors for tumor therapy. In a previous study, TDEs were loaded with miR-375 mimics as vectors and delivered to cancer cells. miR-375-loaded exosomes reduced the migratory and invasive abilities of SW480 and HT-29 CRC cells by reversing EMT (58). siRNA protected by exosomes can also be used for cancer therapy. In head and neck cancer, transfection of FaDu cells with the exosome/TRPP2 siRNA complex increased the expression of E-cadherin and decreased the expression levels of vimentin and N-cadherin, thereby suppressing FaDu cell invasion and metastasis (80).

The behavior of exosomes from different sources can provide new insights into cancer treatment. Exosomes derived from bone marrow mesenchymal stem cells promote tumor development. Exosomes may regulate tumor characteristics by activating the sonic hedgehog signaling pathway. The study of bone marrow mesenchymal stem cell-derived exosomes that activate the Sonic Hedgehog signaling pathway to regulate tumor invasion and metastasis will contribute to revealing tumor pathogenesis and possible therapeutic approaches (81, 82). In addition, a study has shown that CSC-derived exosomes preserve the biological characteristics of CSCs, promoting the proliferation and migration of human umbilical cord mesenchymal stem cells and vascular endothelial cells, and enhancing the resistance of tumor cells to chemotherapy by promoting the expression of tumor-related drug resistance genes. It is possible to discover potential targets for tumor therapy by studying the correlation between tumor stem cell-derived exosomes and drug resistance genes (83, 84). Exosomes as vectors cannot only be used for cancer treatment but also for overcoming drug resistance. LT et al. (78) encapsulated miR-128-3p in FHC cell-derived exosomes and delivered it to oxaliplatin-resistant cells. The inhibited expression of drug transporters reduced the efflux of oxaliplatin and alleviated chemotherapy resistance in colorectal cancer cells. Moreover, oxaliplatin-induced EMT was inhibited by the overexpression of miR-218-3p.

Although the majority of tumor treatment studies have used exosomes to inhibit EMT, a number of studies have attempted to inhibit the production of exosomes and regulate the anti-tumor effects of EMT. TF et al. (85) used cetuximab to prevent the production of exosome vesicles and reduce exosome secretion to induce EMT in oral cancer cells.

In addition to human exosomes, which can be used to treat diseases, plant exosomes can also function in humans across races. In animal experiments, PELNVs secreted by ginger inhibited the expression of the secreted protein hemagglutinin, promoted wound healing, reduced the level of cyclin D1 mRNA in a mouse model of colon cancer, and inhibited the occurrence and development of CRC (86).

## Challenges and Prospects

In this review, we summarize the latest research advancements in the regulation of the EMT process in tumor cells by the contents of exosomes. However, several outstanding issues remain. There are many challenges in this research field. In terms of mechanistic research, the analysis of exosome components is incomplete, and the biogenesis of exosomes and the principle of content screening are unclear. Considering that cancer is a highly heterogeneous disease, the mechanism underlying the different effects of exosomes on EMT in different cancers is still unknown.

The exploration space for research on exosomes as a tool for cancer treatment is broad. At present, the treatment of cancer with exosomes is limited to the laboratory level and has not been applied in clinical practice. In addition, efficient and high-purity exosome isolation technology is lacking, which poses certain difficulties for the detection of cancer-related indicators using exosomes in research and clinical treatment. The relationship between different cell-derived exosomes and their therapeutic effects on EMT is still unknown. Elucidating this problem will help to formulate more effective treatment options. Researchers are also working on the accurate remolding of exosomes. Existing research has indicated that exosomes can be organ-targeted. Whether this feature can accurately regulate cancer progression, including EMT, remains unknown.

Although the treatment of cancer with PELNVs is receiving increased attention, we still have a limited understanding of the process of PELNV internalization by animal cells. Compared with exosomes derived from animals, PELNVs are slightly inferior because of their low toxicity and immunogenicity, which are some of the problems to be solved. Thus, there are many gaps in research on the regulation of EMT by exosomes in cancer. Nevertheless, this research field is promising.

In this review, we summarize the latest research advances in the regulation of the EMT process in tumor cells by the contents of exosomes. Furthermore, we discuss the potential and challenges of using exosomes as a tool for cancer treatment. Determining the mechanisms underlying EMT regulation by exosomes will contribute to designing new therapeutics that target exosome-mediated tumor metastasis and chemoresistance. We hope that our review will help researchers comprehend the relationship between exosomes and EMT and conduct further investigations in this area.

## Author Contributions

JJ and JL have equally contributed to this project. YQ, JJ, and JL have contributed to information interpretation, editing and critical revision of the manuscript. XMZ and XQZ were responsible for drawing the pictures. YQ and BH contributed to study design and critical revision of the manuscript. All authors contributed to the article and approved the submitted version.

## Funding

Major Science and technology projects in Xiaoshan (Project Number: 2020207). Scientific Research Foundation of Zhejiang Sci-Tech University (Project Number: 21042102-Y). Key Research and Development Project of Hangzhou(Project Number:202004A23).

## Conflict of Interest

The authors declare that the research was conducted in the absence of any commercial or financial relationships that could be construed as a potential conflict of interest.

## Publisher’s Note

All claims expressed in this article are solely those of the authors and do not necessarily represent those of their affiliated organizations, or those of the publisher, the editors and the reviewers. Any product that may be evaluated in this article, or claim that may be made by its manufacturer, is not guaranteed or endorsed by the publisher.
